# Pleomorphic Adenoma of the Palate: A Detailed Case Report and Surgical Outcome

**DOI:** 10.7759/cureus.79673

**Published:** 2025-02-26

**Authors:** G. Venkateswara Reddy, G. Siva Prasada Reddy, Mohammed Darain Shahid, Vishnu Priyatham, Sriram Ravali

**Affiliations:** 1 Department of Oral and Maxillofacial Surgery, Panineeya Mahavidyalaya Institute of Dental Sciences and Research Centre, Hyderabad, IND

**Keywords:** head and neck tumors, intraoral mass, palatal tumor, pleomorphic adenoma, pleomorphic adenoma of hard palate, salivary gland tumor, surgical excision

## Abstract

Pleomorphic adenoma is the most common salivary gland neoplasm, accounting for a significant proportion of both major and minor salivary gland tumors. While it most frequently arises in the parotid or submandibular glands, it can occasionally present as an intraoral mass over the palate or lip when originating from the minor salivary glands. A palatal pleomorphic adenoma typically presents as a painless, gradually enlarging mass on the posterior lateral aspect of the palate. This article discusses a case of a palatal pleomorphic adenoma, which was successfully managed with surgical excision.

## Introduction

Pleomorphic adenoma is a benign mixed tumor that includes both epithelial and myoepithelial cells arranged in diverse patterns and is clearly separated from the surrounding tissues by a fibrous capsule [1]. The name "pleomorphic" indicates the varied embryonic origin of these tumors, which contain both epithelial and mesenchymal components, and they arise from intercalated and myoepithelial cells [2-3]. This type of tumor is common in salivary glands, affecting both major and minor glands, and makes up 40-70% of all salivary gland tumors, with the parotid gland being the most frequently affected. Pleomorphic adenoma of the minor salivary gland in the hard palate is an uncommon condition, representing 5-7% of all salivary gland tumor cases. 

Among the minor salivary glands, the palate is the most affected site (42.63%), followed by the lip (10%), buccal mucosa (5.5%), retromolar area (0.7%), and floor of the mouth [4]. Pleomorphic adenoma generally manifests in individuals during their fourth to sixth decades of life and is more prevalent in women (60%) than in men (40%) [5]. Clinically, pleomorphic adenoma of the hard palate typically appears as a firm or rubbery submucosal mass without ulceration or surrounding inflammation, and it is generally not associated with pain or tenderness [6]. Diagnosing pleomorphic adenoma involves a combination of patient history, physical examination, cytology, and histopathology. Imaging techniques, such as computed tomography (CT) and magnetic resonance imaging (MRI), are crucial for assessing the tumor’s location, size, and extent, and for evaluating its impact on surrounding tissues. In this case report, we present a case of a pleomorphic adenoma of the hard palate in a 37-year-old female who underwent surgical excision.

## Case presentation

A 37-year-old female reported to the Department of Oral and Maxillofacial Surgery, Panineeya Mahavidyalaya Institute of Dental Sciences and Research Centre, Hyderabad, India, with a complaint of swelling in the upper right back tooth region for three years.

The patient's history indicated a painless swelling that gradually increased over the course of three years to its current size. The patient had used Ayurvedic medication for the last year; however, the swelling did not resolve and continued to grow. There were no accompanying symptoms, such as numbness, dysphagia, breathing issues, stridor, speech problems, or masticatory difficulties. The patient had no history of trauma, fever, or similar swellings elsewhere in the body. Past medical history revealed that the patient was otherwise healthy, with no systemic diseases or harmful habits.

**Figure 1:**
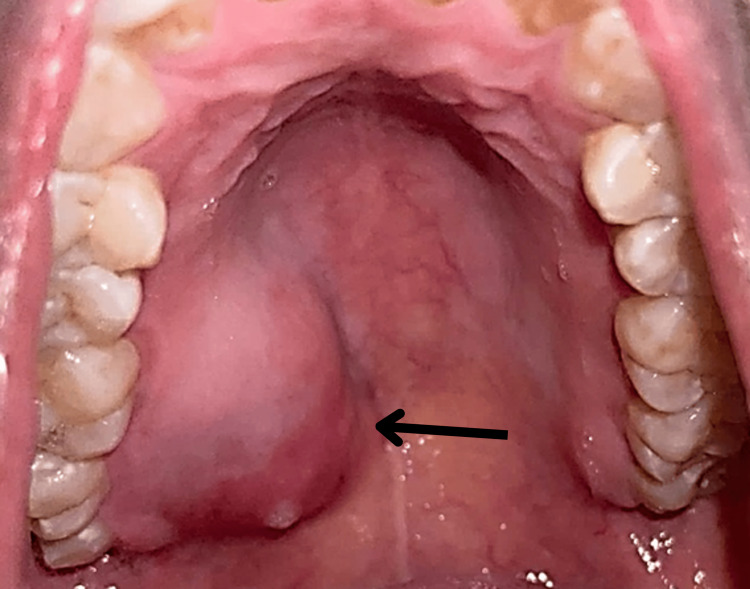
Preoperative intraoral photograph of the patient showing a solitary nodular swelling in the right posterolateral part of the hard palate

During the general physical examination, the patient appeared moderately built and fully conscious and exhibited a normal gait. Her vital signs were stable and within normal limits. An extraoral examination revealed no facial asymmetry or lymphadenopathy. On intra-oral examination, a solitary nodular swelling was seen in the right posterolateral part of the hard palate measuring 4 × 3 cm in size, extending anteroposteriorly from 15 to 18 and medially toward the midline of the hard palate (Figure 1). On palpation, all inspection findings were confirmed. The swelling was firm, non-tender, non-pulsatile, and immovable, with well-defined margins. The overlying mucosa appeared stretched, but intact. Intraoral examination of hard tissues revealed no dental anomalies associated with the lesion.

**Figure 2:**
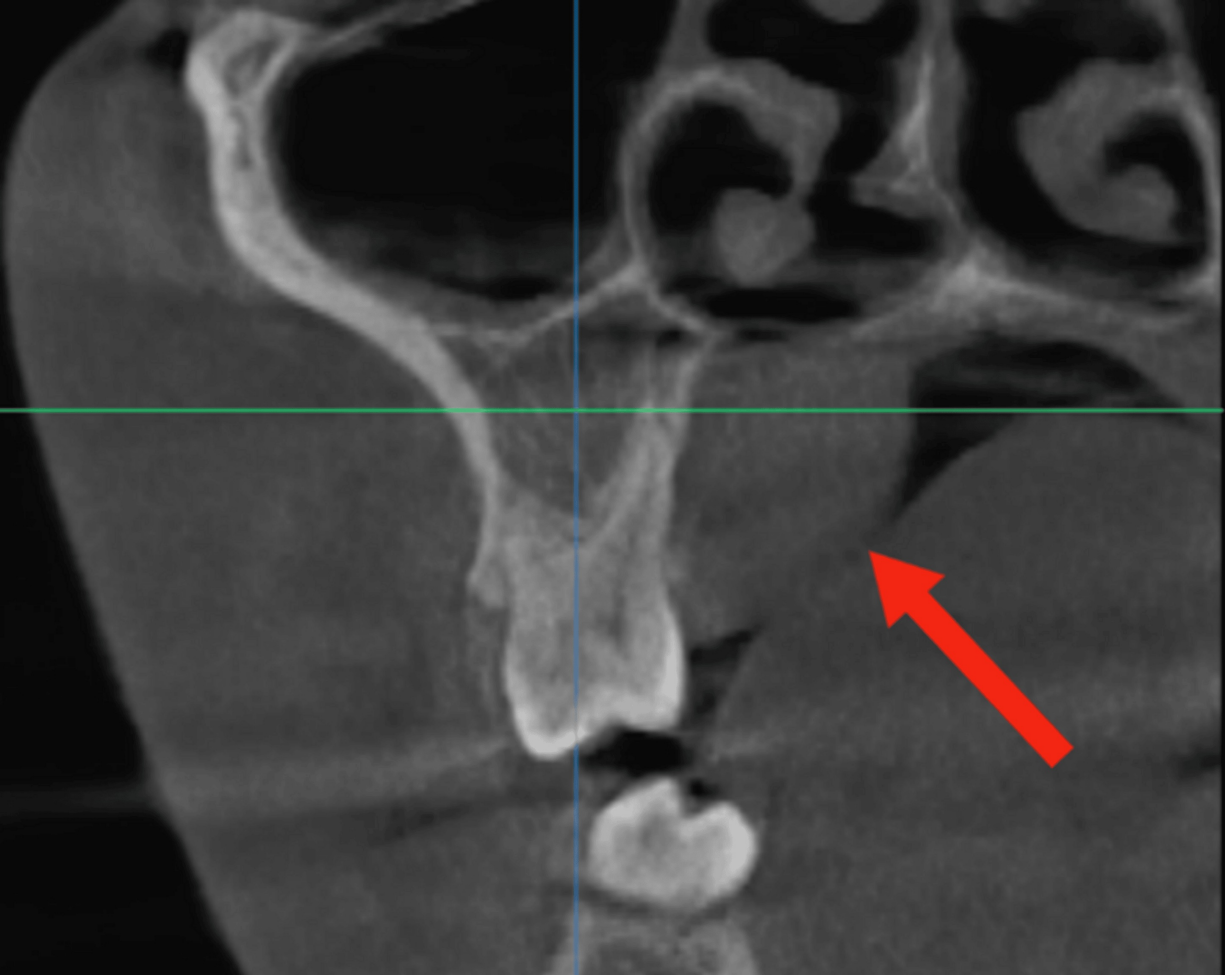
Cone-beam computed tomography (CBCT) section showing an iso-dense structure in the hard palate

The cone-beam computed tomography (CBCT) of the patient revealed an iso-dense structure in the palate with no signs of bony erosion of the palatal region (Figure 2).

**Figure 3:**
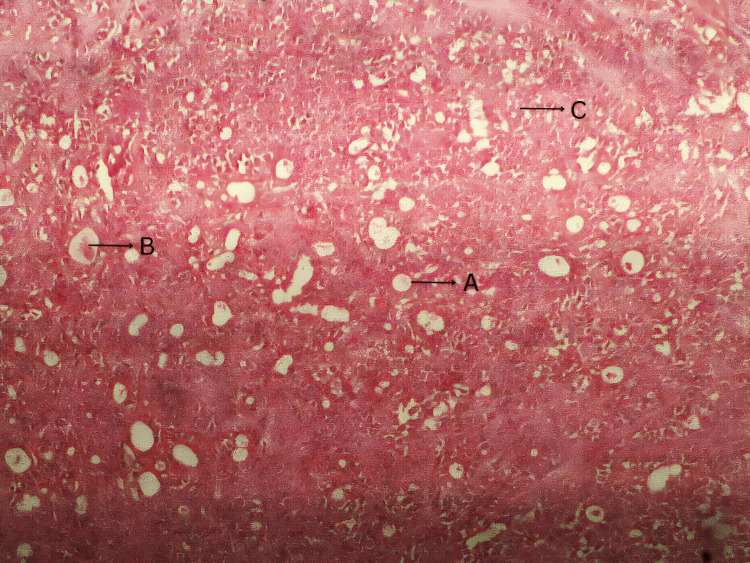
Photomicrograph revealing A) epithelial cell arranged in the form of a duct, B) eosinophilic coagulum in the center of the epithelial duct, and C) connective tissue cell resembling a myoepithelial cell

Accordingly, we opted for an incisional biopsy under local anesthesia, and the excised specimen was sent for histopathological examination. The photomicrograph (Figure 3) of the mass revealed epithelial cells arranged in the form of islands, sheets, and ducts. The central portion of epithelial ducts had an eosinophilic coagulum. The background connective tissue showed angular and spindle-shaped cells resembling myoepithelial cells along with adipocytes at focal areas. This confirmed the diagnosis of pleomorphic adenoma.

**Figure 4:**
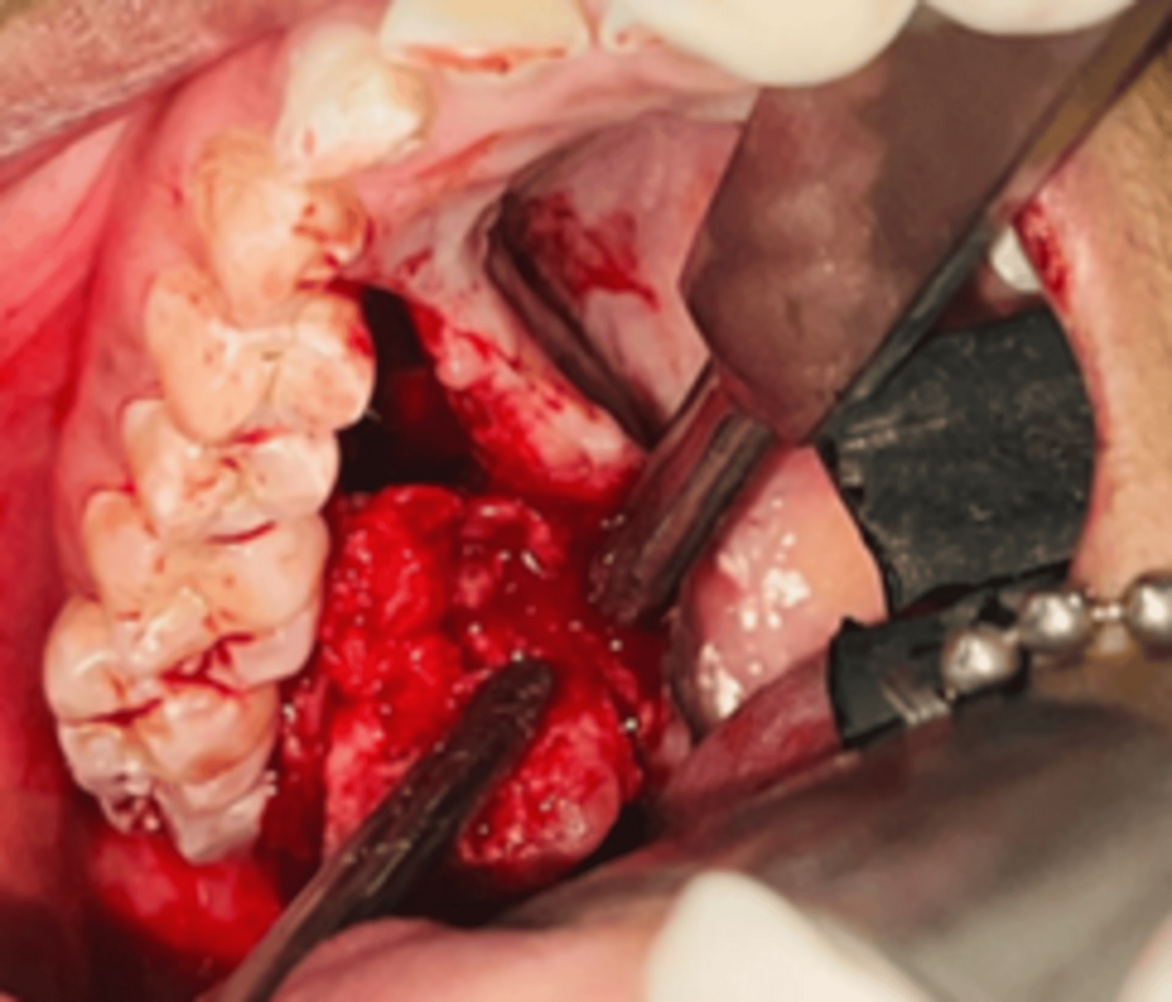
Intraoperative picture of the lesion being excised

After routine preoperative investigations, the case was planned for surgical excision under general anesthesia. Under sterile aseptic conditions, local infiltrations were administered using 2% lignocaine with a 1:80,000 dilution of adrenaline. A crevicular incision was made, extending from the distal aspect of tooth 13 to tooth 18, while a releasing incision was made distal to tooth 18. A full-thickness mucoperiosteal flap was raised, revealing that the tumor was attached to the overlying palatal mucosa. Tissue dissection was performed to separate the tumor from the palatal mucosa. During the procedure, it was observed that the tumor encased the greater palatine neurovascular bundle. Consequently, it was ligated, and the tumor was excised (Figure 4).

**Figure 5:**
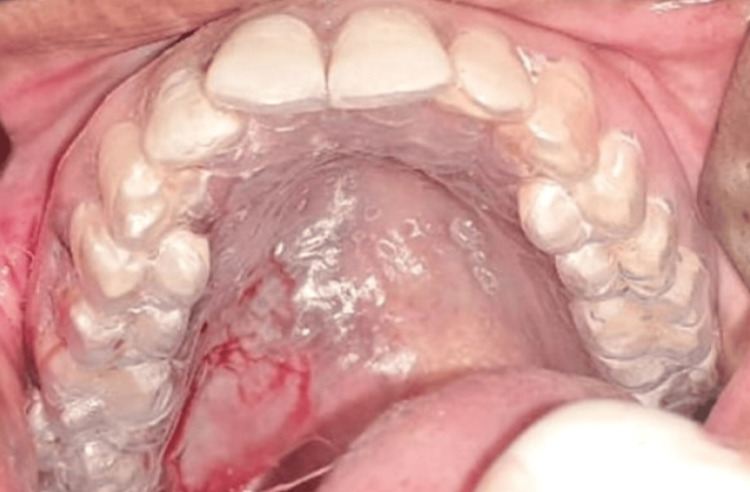
Postoperative intraoral photograph with a maxillary splint

**Figure 6:**
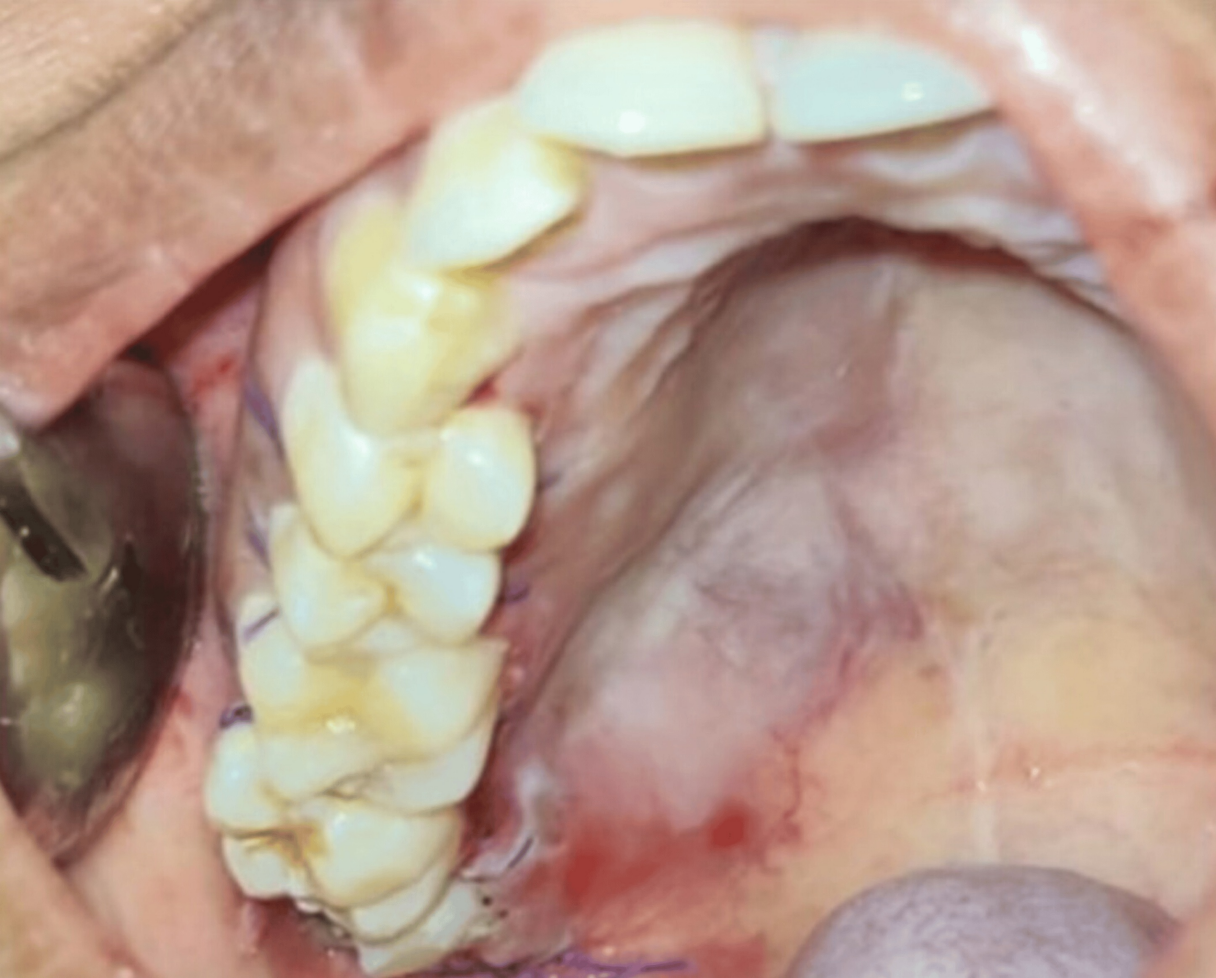
Postoperative intraoral photograph showing good healing of the surgical site after two weeks

**Figure 7:**
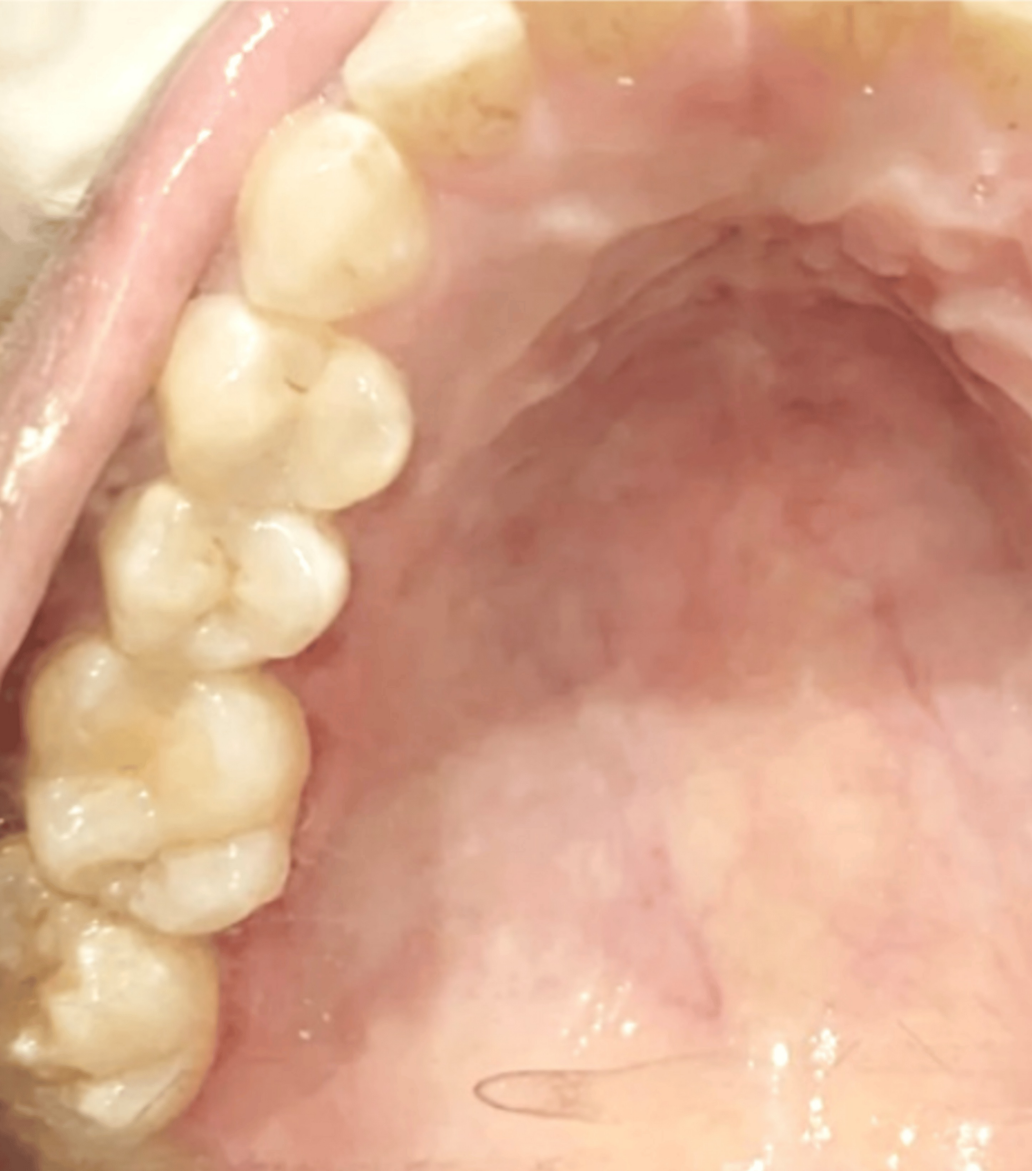
Postoperative intraoral photograph after one year

Hemostasis was achieved and closure was done using 3-0 Vicryl sutures. A maxillary splint that was prefabricated before the surgical excision was subsequently placed (Figure 5). The postoperative period was uneventful, and the maxillary splint was removed two weeks postoperatively when healing was found to be satisfactory (Figure 6). There were no signs of recurrence observed at the one-year follow-up (Figure 7).

## Discussion

Pleomorphic adenoma of the hard palate is a rare entity, accounting for 5-7% of all salivary gland tumors [7]. Clinically, it presents as a painless, slow-growing mass, often delaying diagnosis. In this case, the patient exhibited the classic presentation of a gradually enlarging, painless mass over three years. There were no atypical features such as rapid growth, ulceration, tenderness, or secondary complications that could suggest infection or malignant transformation. However, cases with rapid enlargement or pain require careful evaluation for potential malignant changes.

Imaging modalities such as MRI and CT are invaluable for assessing tumor extent and planning surgical intervention [8]. In our case, CBCT was used as the primary imaging modality to evaluate the lesion. The scan confirmed a well-defined, iso-dense structure without signs of cortical erosion or bone invasion. This finding suggested a benign nature and guided the surgical approach, allowing for a conservative yet complete excision. While MRI provides superior soft tissue contrast, it was not deemed necessary in this case due to the lesion’s well-demarcated appearance on CBCT.

Histopathological analysis plays a crucial role in confirming the diagnosis of pleomorphic adenoma. Classical pleomorphic adenoma consists of a mixture of epithelial, myoepithelial, and stromal components within a myxoid, chondroid, or fibrous background. The epithelium often forms ducts and cystic structures or may occur as islands or sheets of cells [9]. In our case, histopathology revealed epithelial cells arranged in sheets, islands, and duct-like structures, with myoepithelial cells present in the background. The presence of an eosinophilic coagulum within the ductal lumina was also noted. These findings were consistent with classical descriptions, confirming the diagnosis of pleomorphic adenoma. No atypical cellular features or malignant transformation were observed.

Surgical excision with clear margins is the cornerstone of treatment, as incomplete removal is the primary cause of recurrence, which can occur years after initial treatment [10]. In this case, the tumor’s close proximity to the greater palatine neurovascular bundle presented a surgical challenge. The vessel was ligated to facilitate excision, and careful dissection was performed to avoid excessive bleeding while ensuring complete tumor removal. A prefabricated maxillary splint was used postoperatively to aid healing. Although at the one-year follow-up, there were no signs of recurrence, a longer follow-up period is essential, as pleomorphic adenoma has the potential for late recurrence. While malignant transformation is rare, it underscores the importance of long-term surveillance [11].

## Conclusions

Pleomorphic adenoma of the hard palate, although rare, should be considered in the differential diagnosis of palatal masses. Comprehensive diagnostic evaluation, including imaging and histopathology, is essential for accurate diagnosis. Complete surgical excision remains the gold standard for treatment, with long-term follow-up recommended to monitor for recurrence or malignant transformation.
